# Process mining to discover patterns in patient outcomes in a Psychological Therapies Service

**DOI:** 10.1007/s10729-023-09641-8

**Published:** 2023-05-16

**Authors:** C. Potts, R. R. Bond, J-A. Jordan, M. D. Mulvenna, K. Dyer, A. Moorhead, A. Elliott

**Affiliations:** 1 School of Psychology, Faculty of Life and Health Sciences, Ulster University, Coleraine, Northern Ireland; 2 School of Computing, Faculty of Computing Engineering & the Built Environment, Ulster University, Belfast, Northern Ireland; 3 IMPACT Research Centre, Northern Health and Social Care Trust, Antrim, Northern Ireland; 4 Psychological Therapies Service, Northern Health and Social Care Trust, Antrim, Northern Ireland; 5 School of Communication and Media, Institute of Nursing and Health Research, Ulster University, Belfast, Northern Ireland

**Keywords:** Process mining, Mental health, Therapy, Data analytics, Psychological therapies

## Abstract

In the mental health sector, Psychological Therapies face numerous challenges including ambiguities over the client and service factors that are linked to unfavourable outcomes. Better understanding of these factors can contribute to effective and efficient use of resources within the Service. In this study, process mining was applied to data from the Northern Health and Social Care Trust Psychological Therapies Service (NHSCT PTS). The aim was to explore how psychological distress severity pre-therapy and attendance factors relate to outcomes and how clinicians can use that information to improve the service. Data included therapy episodes (*N* = 2,933) from the NHSCT PTS for adults with a range of mental health difficulties. Data were analysed using Define-Measure-Analyse model with process mining. Results found that around 11% of clients had pre-therapy psychological distress scores below the clinical cut-off and thus these individuals were unlikely to significantly improve. Clients with fewer cancelled or missed appointments were more likely to significantly improve post-therapy. Pre-therapy psychological distress scores could be a useful factor to consider at assessment for estimating therapy duration, as those with higher scores typically require more sessions. This study concludes that process mining is useful in health services such as NHSCT PTS to provide information to inform caseload planning, service management and resource allocation, with the potential to improve client’s health outcomes.

## Highlights

- First study to apply process mining to data from psychological therapies
- Process mining used to understand patterns in client engagement with therapy
- Attendance and missed sessions explored in relation to client outcomes
- A series of policy and practice recommendations for clinicians have been proposed

## Introduction

Mental health problems are one of the main causes of overall disease burden in the United Kingdom (UK) and it is estimated that one in five adults in England have experienced a common mental disorder such as depression and anxiety [1]. This figure is even higher in Northern Ireland, as a recent survey by the Department of Health found that one in five adults showed signs of a mental health problem [2]. Northern Ireland experienced decades of civil conflict and the effects of this still remain today with high prevalence of anxiety, mood or impulse-control disorders in those who experienced the conflict [3]. In addition, an increase in psychological distress has recently been reported among those with pre-existing mental health problems [4] and the general population [5] resulting from the COVID-19 pandemic. This further highlights the need for support, and more importantly, the predicted increased demand in services that is expected to come [6].

Mental Health services that offer psychological therapy are provided by the National Health Service (NHS) across the UK and are structured according to an initiative termed ‘Improving Access to Psychological Therapies’ (IAPT) [7, 8]. IAPT services are provided in England and closely replicated by ‘Psychological Therapies Services’ in Northern Ireland. These services provide National Institute for Health and Care Excellence (NICE) recommended evidence-based interventions [9] for example, Cognitive Behavioural Therapy (CBT) which has been shown to be just as effective as non-directive counselling for mixed anxiety and depression and also for depression alone [10]. Services are structured according to a stepped-care model [11] so a clinical decision can be made as to the type of treatment that is the most appropriate for the individual. Stepped care ranges from ‘Step 1’ which involves support that can be utilised before approaching either health or social services, to ‘Step 5’ for high intensity therapies with specialists [11]. IAPT clients would be transferred or referred between services whereas clients are stepped up or down in the Psychological Therapies Services in Northern Ireland. Psychological Therapies utilise Routine Outcome Measurement (ROM), an evidence-based quality assessment tool that involves routinely monitoring clients presenting difficulties, functioning and progress towards their goals over the course of treatment, usually through the completion of psychometrics [12, 13]. Two key performance indicators of ROM used by IAPT and Psychological Therapies Services in Northern Ireland include: ‘reliable improvement’ (improvement greater than the measurement error of a psychometric scale) and ‘reliable recovery’ (incorporating ‘reliable improvement’ and psychometric scores falling below clinical cut-off post treatment) [14].

Psychological therapies services face a number of challenges with service factors, such as increased demand and associated waiting lists, as well as client factors, for example missed appointments. These factors can significantly impact on service delivery and therapy outcome [15]. Understanding such factors could help services perform better, by assigning clients to a more appropriate intervention; reducing misuse of therapy resources; expediting wait times; and ultimately improving the outcomes of therapy for clients. There is also a lack of consistent data collection and analysis across services which makes it difficult for services to explore these aspects to understand service performance [14]. A ‘therapy session’ is defined as a single individual or group meeting between a client and a mental health professional, whereas a ‘therapy episode’ is a collection of therapy sessions throughout a client's duration with a mental health service. A pilot study found that patient age, gender or overall attitude towards therapy did not affect attendance in a client’s first therapy session [16]. Other studies have looked at predicting patient non-attendance and engagement in IAPT services using statistical modelling of client records [17, 18]. Di Bona et al*.* [17] found that clinical characteristics such as risk to self, severity of emotional distress, and illness duration, along with site, were more predictive of first therapy session non-attendance than socio-demographic characteristics. Davis et al*.* [18] found that those who self-referred were more likely to attend their initial appointments and those who were older, had fewer previous referrals and consented to receiving a reminder message via SMS were more likely to attend their first therapy session. Explication of the precise risk factors likely to affect therapy sessions is useful for predicting missed appointments and outcomes, allowing services to better plan and maximise resources.

In general, research is shifting in the direction of ‘big data’ practice-based evidence which is arguably more useful than traditional evidence-based practice methods like randomised control trials. Analytics approaches using large volumes of psychological therapies outcomes data provide greater clarity on what contributes to successful therapy and reduction of client mental ill health symptoms [14, 19]. Data science, which can be described as generating new knowledge by analysing real-world data [20], can be used to provide valuable insights into therapy services. One such approach, process mining, has already been successfully applied across different areas including business and IT and more recently in the healthcare domain. Process mining aims to discover, monitor and improve real processes by extracting knowledge from event logs [21]. Event log data typically consists of an anonymous label assigned to each case (unique identifier), a start and end date-time for each case (date-time stamp) and activity (for example in a healthcare setting this could be registration, triage, x-ray, discharge) but may also include other contextual factors. The digitalisation of healthcare has led to the development of Healthcare Information Systems (HIS) that collect and analyse data from the healthcare sector, ensure their overall quality, relevance and timeliness, and convert data into information for health-related decision-making [22]. These systems store patient level event logs, including electronic health records which are amenable to process mining. The three main types of process mining are discovery, conformance, and enhancement. Discovery focuses on the inference of process models from the event logs with no prior information [23]. Conformance involves comparing an existing process model to event logs from the same process [23]. Conformance checking can be used to assess if the event logs conform to the model and vice versa [24]. Enhancement consists of extending or improving an existing process model using the event logs to obtain information about the process itself [23].

The main applications of process mining in healthcare to date as identified by Rojas et al*.* [25] have been to effectively manage the flow of hospital patients [26, 27], reveal the order of clinical activities [27, 28], identify bottlenecks [29, 30], identify outliers when compared against a theoretical workflow model [31], and examine the relationship between resources [32, 33]. A recent review by Halawa et al*.* looked at advancing healthcare based on evidence-based research [34]. This included studies that utilised process mining to analyse hospital patient tracks to reduce distances traveled by patients [35] and improve hospital layout design [36]. The authors noted that the main finding after analysing these studies was that no one methodology suits all, and that multiple approaches providing a solid framework along with comparing various process mining and machine learning algorithms should be considered for the future [34].

Most of the previous applications of process mining in healthcare have been in a hospital setting and to the best of our knowledge, process mining has not been applied to a mental health service to date. Valuable insights can be gained through innovative mining of psychological therapies data to discover processes and how they relate to client outcomes. This study set out to address the following questions: for clinicians and practitioners, what key insights can be gained from process mining of Northern Heath and Social Care Trust Psychological Therapies (NHSCT PTS) data? What policy and practice recommendations can be made to the NHSCT PTS based on findings from process mining? The overall aim of this study was to apply process mining to explore how pre-therapy psychological distress severity and attendance factors relate to outcomes and how clinicians can use that information to improve the NHSCT PTS service.

## Methods

### Research design

This research was a data analytics study using process mining. Overall, the process mining methodology can be broken down into multiple stages including: defining the project, preparing the data, analysing processes, and redesigning processes [37]. The initial phase where the project is defined typically involves describing the problem and determining the project objectives or questions. Next the data is prepared, which includes locating and extracting the data, analysing the quality, data cleaning and carrying out any additional data preparations or transformations [37]. Process mining can then be undertaken to discover the real process models, analyse their performance, establish the interactions between the people involved in the process and verify if the processes follow service procedures [37]. The final stage looks at using the results of process mining to determine if improvements can be made, along with evaluating and implementing alternatives and measuring the results [37]. The “patient process flow” for this study included events that took place throughout the client's journey with PTS, from their initial appointment, including events that occurred during therapy, to their final appointment with the service. The event logs included information captured about sessions with the aim of discovering information that could improve services. This study has obtained ethical approval from the School of Communication & Media Filter (Ethics) Committee, Ulster University, and research governance from the Northern Health and Social Care Trust, Northern Ireland.

### Data provenance and participants

Data in this study were obtained from the NHSCT PTS, a trust wide service in Northern Ireland for adults with a range of mental health difficulties including anxiety disorders such as phobias, panic disorder, social phobia, obsessive compulsive disorders, post-traumatic stress disorder, anxiety and depression. The service also offers psychological interventions for more severe and enduring problems. The NHSCT PTS staff includes Clinical and Counselling Psychologists, Cognitive Behaviour Therapists, Psychological Therapists, Associate Psychologists and Assistant Psychologists. Staff offer consultation, one-to-one treatment, group work, brief interventions, supported self-help and computer assisted treatments. The NHSCT PTS has a large database detailing ROMs which has allowed the service to assess improvement following psychological therapy and to gather feedback from service users.

Data on service users are routinely collected via paper records which are subsequently entered into a database by staff at the NHSCT PTS. An anonymised version of the database (i.e. excluding patient’s Health and Care number and local database identifier) was used in the present research. The version used covered the period from September 2007 to September 2019 and included an anonymous unique identifier variable thus allowing for individual and episode based analyses. Each row of the database is defined as a ‘therapy episode’. An episode is essentially the journey of a client through the service, from initial assessment to their last session. In some cases, individuals present to the service more than once and will therefore have multiple ‘therapy episodes’. The present study explored therapy episodes and thus the results presented are characteristics of each episode rather than individual clients.

### Data analysis

The methodology for this study follows a similar approach to that proposed by Graafmans et al. [38], which combines the Define-Measure-Analyse-Improve-Control (DMAIC) model with process mining. The present study utilises only the Define-Measure-Analyse aspect of the model [38]. The “define” phase included planning (establishing research questions), initial preparation of the data (data wrangling) and exploratory data analysis. The “measure” phase involved further exploratory analysis, transforming the data into event logs and carrying out process mining using selected features in the data. The “analyse” phase, involved trying to identify opportunities for improvements in the service. R programming language and RStudio (version 3.6.0) were used for all data analyses.

### Data wrangling

Data were filtered to include the following: gender, age, individual unique identifier, first and last therapy date, pre and post psychological distress severity bands, number of individual sessions attended, number of group sessions attended, total number of individual and group sessions attended, CNAs (Could Not Attend), DNAs (Did Not Attend), psychological distress change category, and nature of discharge. Rows with one or more missing values on these variables were omitted for all models. Numerical data were binned into categories, as process mining requires categorical data.

### Features

The final models included the following features:

#### Pre and post psychological distress – severity band

Psychological distress was measured by the Clinical Outcomes in Routine Evaluation (CORE). The CORE outcome measure is a widely-used general psychometric measure of psychological distress, tapping dimensions of wellbeing, functioning, problems and risk. CORE has previously demonstrated strong internal consistency (0.82 -0.90) [39, 40]. Several versions are available, including the original 34-item version (CORE-OM) [39] and a briefer 10-item version (CORE-10) [40] with scope to transform scores making comparison valid between the measures. CORE-10 has high internal reliability (0.7), is highly correlated with the CORE-OM (> 0.9) and has been validated for screening in primary care patients as well as the general population [40]. NHSCT PTS therapists administer the CORE-OM at the start and end of therapy, while the CORE-10 is administered at every session in between. In the NHSCT PTS database, all CORE-OM scores are transformed to CORE-10, so all scores are recorded the same way to allow easy comparison (scale range 0–40). Only the CORE scores at the start and end of therapy are recorded electronically in the database. If an individual dropped out of therapy before the planned end date, then the CORE-10 score from their last session was used as their post-CORE score. These scores were separated into bands based on CORE documentation [41] as follows; severe (25 to 40), moderately severe (20 to < 25), moderate (15 to < 20), mild (10 to < 15), low (6 to < 10), healthy (0 to < 6).

#### Pre-post psychological distress—change category

The Reliable Change Index (RCI) [40] was used to measure change in CORE scores from the beginning to end of therapy. Pre and post-CORE score differentials were coded as follows: significant improvement—decreased by 6 points or more; non-significant improvement—decreased by fewer than 6 points; significant deterioration—increased by 6 points or more; non-significant deterioration—increased by fewer than 6 points.

#### Nature of discharge

This feature is a clinician based assessment of the individual in terms of whether therapy goals were achieved or if a client self-discharged before the planned therapy end date. Nature of discharge is divided into four categories; goals achieved, goals partially achieved, no change and dropped out of therapy.

#### Could not attend (CNAs)

Number of sessions missed because the client cancelled.

#### Did not attend (DNAs)

Number of sessions that were not attended, where no prior notice was given.

#### Number of sessions

The total number of individual and group sessions attended by the client were categorised based on exploratory analysis and knowledge of NHSCT PTS. “Step” allocation was used to help justify the boundaries for sessions. At initial assessment clients were allocated to a step of the Stepped Care Model similar to the approach used by IAPT services [9], depending on the complexity and severity of their presentation. Step allocation is usually associated with, but not prescriptive of, intervention duration. Typical durations for each step allocation are: Step 2 (“mild”): 6–8 sessions, Step 3 (“moderate”): 8–15 sessions, Step 4 (“severe”): 16–20 sessions, Step 5 (“very severe”): 20 + sessions. The total number of sessions attended were categorised via exploratory analysis into four groups which are broadly in line with duration patterns seen within step allocations, specifically: 2—7 sessions, 8—15 sessions, 16—20 sessions, > 20 sessions.

### Descriptive statistical analysis

The dataset was explored which involved plotting histograms and boxplots for age, total number of sessions, individual sessions, group sessions, CNAs, DNAs and both pre- and post-therapy scores for psychological distress. Barplots were produced for gender. Total number of sessions, individual sessions, group sessions, CNAs, DNAs and post-therapy psychological distress score were also visualised across each pre-therapy psychological distress severity band. Each feature was assessed for normality using a Shapiro–Wilk test. For all features, *p* < 0.05 indicated data were not normally distributed. This was confirmed by visual inspection of the histograms/ boxplots, indicating that non-parametric testing should be applied. Kruskal–Wallis tests were performed for age, total number of sessions, individual sessions, group sessions, CNAs, DNAs, and post-therapy psychological distress scores across each pre-therapy psychological distress band (alpha = 0.05). The null hypothesis of the Kruskal–Wallis test is that the mean ranks of the groups are the same and the alternative hypothesis is that the mean ranks of the groups are different. Post-hoc analysis was carried out using pairwise Wilcoxon Rank Sum Tests with Bonferroni correction for multiple testing. Bonferroni adjustments were applied given they are commonly used due to their simplicity. However, it is worth pointing out they are only one tool that can be used to address the multiple testing problem and this approach yields slightly lower power compared to the other methods for complete data. A chi-squared test was used to compare the proportions of gender across the pre-therapy psychological distress severity bands along with pairwise comparisons with Bonferroni correction for multiple testing. The null hypothesis is that there are no relationships between gender and pre-therapy psychological distress severity, and the alternative hypothesis is that there is a relationship between the two. Numerical features were binned into categories as outlined above.

### Process mining

From the initial data, eight datasets were created for the different aspects of therapy, each containing 2,933 rows (therapy episodes). These included 1. Pre psychological distress – severity band; 2. Total number of therapy sessions; 3. Therapy sessions broken down into individual and group sessions; 4. Missed appointments (DNAs); 5. Missed appointments (CNAs); 6. Post psychological distress – severity band; 7. Pre-post psychological distress—change category; 8. Nature of discharge. Each dataset contained the same features, including the anonymous identifier for each client, appointment date, appointment type (first, during therapy or last) and ‘activity’ which was specific to the categories for each of the eight datasets. The R package BupaR was used for process mining and map visualisation [42] and processcheckR was used for conformance checking. The datasets were converted to event logs as follows:

- case_id = anonymous identifier for each client
- activity_id = range of activities
- activity_instance_id = unique row number
- lifecycle_id = identifier for ‘first session’, ‘during therapy’ or ‘last session’ to indicate activities that took place at the start of therapy, during therapy and end of therapy
- timestamp = appointment dates. The ‘first’ date was the date of the first therapy session, and the ‘last’ date was the date of the final therapy session. For events which took place during therapy, such as missing appointments, an additional ‘during therapy’ date was generated as the BupaR package requires dates and no missing values to run process mining. The ‘during therapy’ date was input as + 5 days after the first therapy session.

Process maps were initially created for only two features, beginning with the pre-therapy psychological distress band and terminating with one outcome measure: post-therapy psychological distress severity band, post-therapy psychological distress change category, or nature of discharge. For the maps which included psychological distress severity band as an outcome, additional maps were produced including total number of sessions, DNAs and CNAs as intermediary variables. Processes were explored for all clients but due to the complexity and readability of diagrams, only a subset of data were visualised as process maps. All process maps shown represent at least 80% or more of all clients and are intended to capture the majority of pathways through the service. The percentage of clients shown is indicated in the figure legends. For example, where it states “90% of episodes” this means approximately 90% of the cases that share the most common traces (pathways). Using the trace explorer function in bupaR, the clients that took the least common pathways were investigated and the results described within the text. ProcesscheckR was also used to explore differences between the process maps that were modeled and what was observed in the data.

**Fig. 1:**
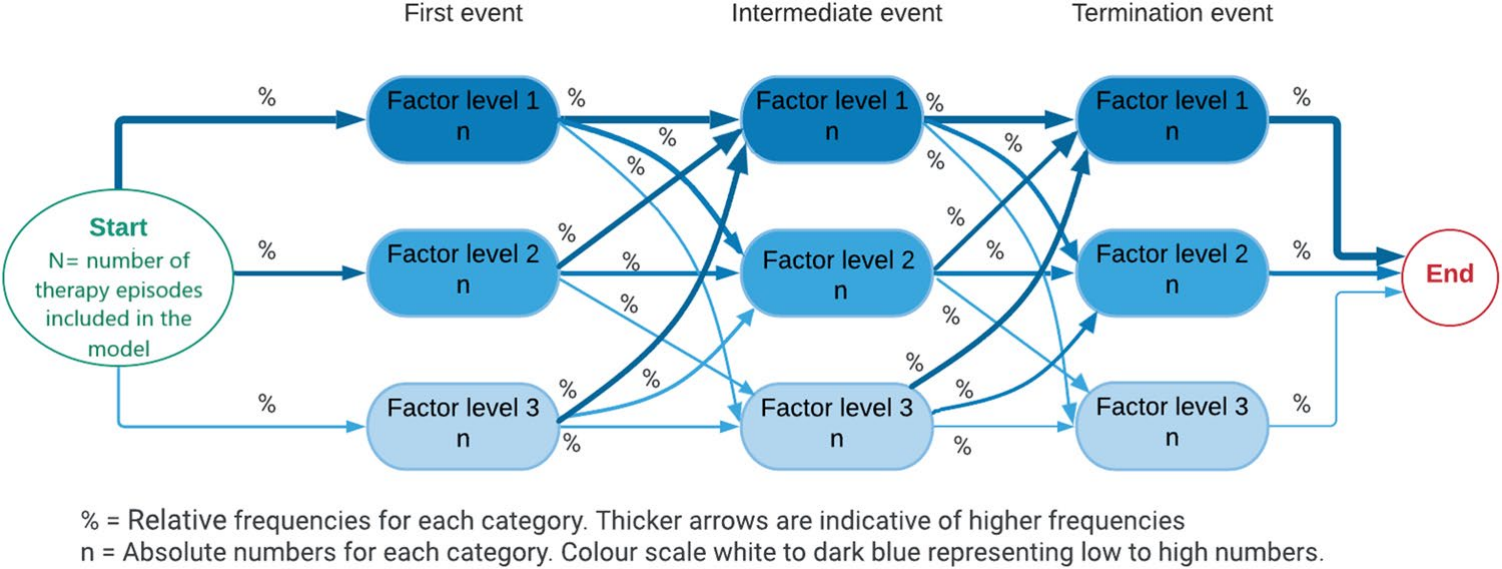
Overview of visualisation method for process maps

In this study first level events are those that take place at the beginning of therapy, intermediate events take place during therapy and termination events at the end of therapy (Fig. 1).

## Results

### Service Users

A total of 2,933 therapy episodes from the psychological therapies service were analysed, which included data from 2,766 unique individuals. The majority of these clients used the NHSCT PTS service once (n = 2,605, 94.2%), while 155 people (5.6%) had used the service twice and 6 people (0.2%) had used the service 3 times.

### Psychological Distress

Data were explored across pre-therapy psychological distress bands for different characteristics including age, gender and information on sessions (Table 1). Overall, clients ranged from 18–89 years of age, with an mean age of 41.2 (SD 13.7) and median age of 41.0 (IQR 21). Approximately 60% of therapy episodes were attended by those who identify as female, while the other 40% were attended by individuals who identify as males. There was no statistically significant difference in gender or number of group sessions across pre-therapy psychological distress bands while age, number of individual sessions, CNAs, DNAs and number of sessions were all statistically significant (*p* < 0.001) across groups (Table 1). Generally, those who began therapy in the ‘severe’ psychological distress category were slightly older on average compared to those in the other categories (*p* < 0.001, Table 1). Post-hoc pairwise comparisons showed statistically significant differences in age between pre-severe compared to pre-mild, pre-moderate, and pre-low (*p* < 0.05 for all) psychological distress, while the other comparisons were not statistically significant. Generally, those who had higher psychological distress scores at the beginning of therapy had more individual sessions, CNAs, and DNAs (*p* < 0.001, Table 1). All pairwise comparisons were statistically significant when comparing the number of individual sessions across pre-therapy psychological distress bands, except for bands that were immediately succeeding each other (i.e. comparisons between pre-low and pre-healthy etc. were non-significant). Post-hoc pairwise comparisons showed statistically significant differences in CNAs between pre-severe compared to pre-healthy, pre-low, pre-mild, pre-moderate, pre-moderately severe (*p* < 0.05 for all) while the other comparisons were not statistically significant. Post-hoc pairwise comparisons showed statistically significant differences in DNAs between pre-severe compared to all other pre-therapy psychological distress categories (*p* < 0.05 for all), and similarly for pre-moderately severe compared to all other pre-therapy psychological distress bands while the other comparisons were not statistically significant.

**Table 1:** Characteristics of psychological therapies service episodes (*N* = 2,933)

		Pre-therapy psychological distress (CORE) therapy score						P-value
		Healthy (*n* = 129)	Low-level (*n* = 212)	Mild (*n* = 431)	Moderate (*n* = 604)	Moderately severe (*n* = 718)	Severe (*n* = 839)	
Age (years)	Range	18–78	18–89	18–87	18–88	17–78	18–81	
	Mean (SD)	40.9 (15.4)	41.5 (15.2)	39.8 (14.4)	41.0 (14.4)	40.5 (13.4)	42.5 (12.2)	< 0.001^a^
	Median (IQR)	38 (22)	39 (22.3)	37 (20)	40 (21)	41 (22)	43 (18.5)	
Gender	Male-identifying, n (%)	52 (40.3)	77 (36.3)	167 (38.7)	225 (37.2)	271 (37.7)	337 (40.2)	0.83^b^
	Female-identifying, n (%)	77 (59.7)	135 (63.7)	264 (61.3)	379 (62.8)	447 (62.3)	502 (59.8)	
Individual sessions	Range	0–37	0–58	0–50	0–63	0–96	0–120	< 0.001^a^
	Mean (SD)	9.9 (6.7)	10.8 (7.7)	11.5 (7.4)	12.4 (8.2)	14.3 (11.2)	16.2 (13.8)	
Group sessions	Range	0–25	0–19	0–22	0–23	0–55	0–36	0.27^a^
	Mean (SD)	0.9 (3.3)	0.7 (2.7)	0.8 (3.0)	1.1 (3.6)	0.9 (4.1)	0.8 (3.2)	
CNAs	Range	0–7	0–9	0–11	0–13	0–22	0–18	< 0.001^a^
	Mean (SD)	1.2 (1.5)	1.5 (1.7)	1.3 (1.8)	1.4 (1.8)	1.7 (2.1)	2 (2.3)	
DNAs	Range	0–5	0–8	0–13	0–13	0–21	0–19	< 0.001^a^
	Mean (SD)	0.7 (1.1)	1.0 (1.4)	1.1 (1.9)	1.2 (1.6)	1.7 (2.4)	2.2 (2.6)	

^a^Kruskal Wallis test p-value

^b^Chi-squared test p-value

### Process Mining

**Fig. 2:**
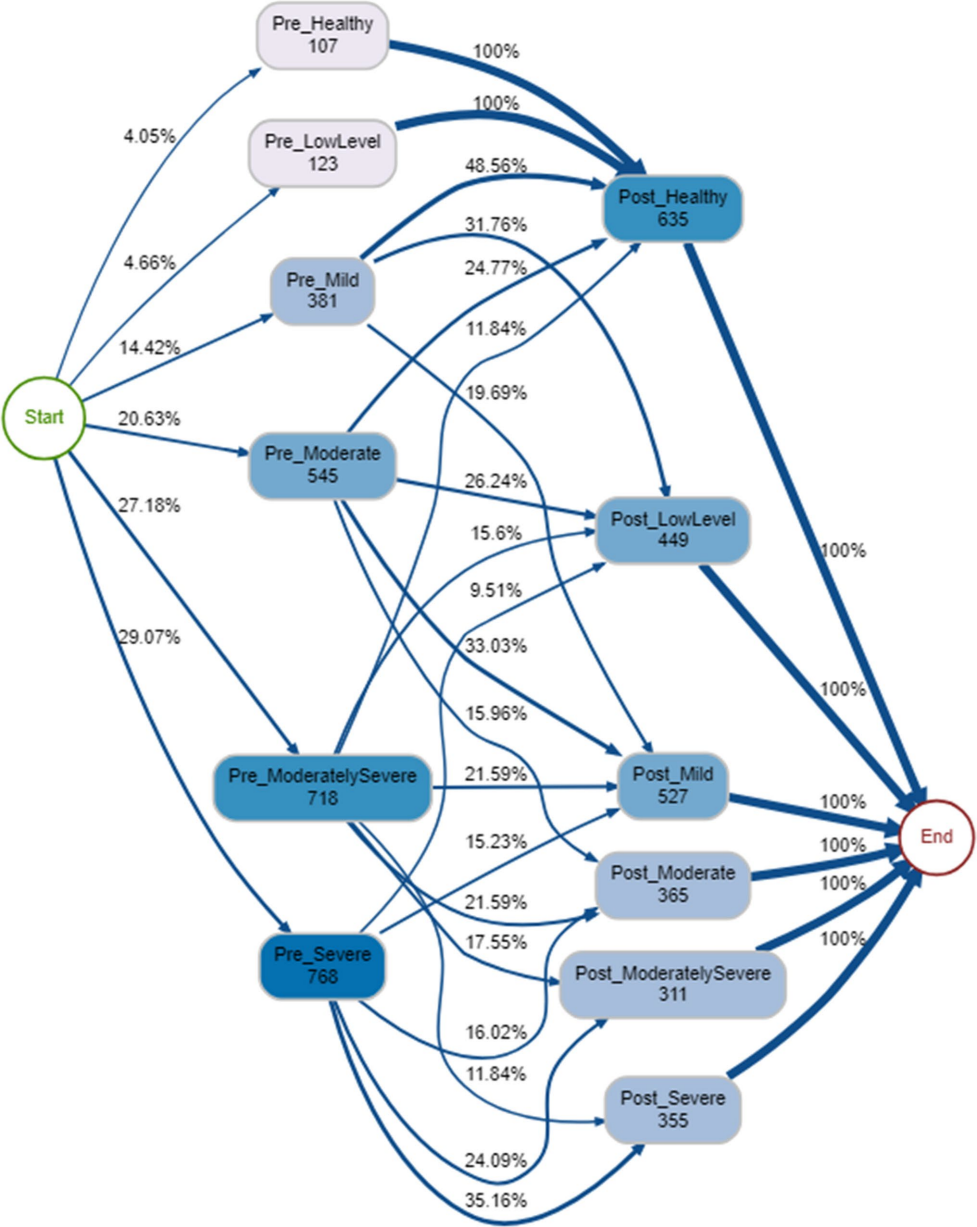
Process map for pre-therapy psychological distress severity band → post-therapy psychological distress severity band (90% therapy episodes shown)

For the majority (90%) of therapy episodes, the psychological distress band at the beginning (‘Pre’) was largely determinant of the psychological distress band at discharge (‘Post’), as clients tended not to change up or down by more than one band (Fig. 2). In a model accounting for 90% of episodes (Fig. 2), all of those that started off in the ‘healthy’ band remained in the same band at the end of the therapy and all those who started off as ‘low’ improved to ‘healthy’. However, several edge cases were identified for therapy episodes which did not comply with these rules. A small number of individuals (*n* = 21) who started therapy in the ‘healthy’ band ended therapy in a higher band (low level, mid, moderate) and one individual ended therapy with ‘moderately severe’ psychological distress. Using the model shown in Fig. 2, 35% of those that started therapy with severe psychological distress ended therapy in the same band (Fig. 2). Overall, the most common single pathway through the service was pre-severe → post-severe (*n* = 270, 9.2%). The least common pathways included those who went through the service and ended in a more severe band post-therapy.

**Fig. 3:**
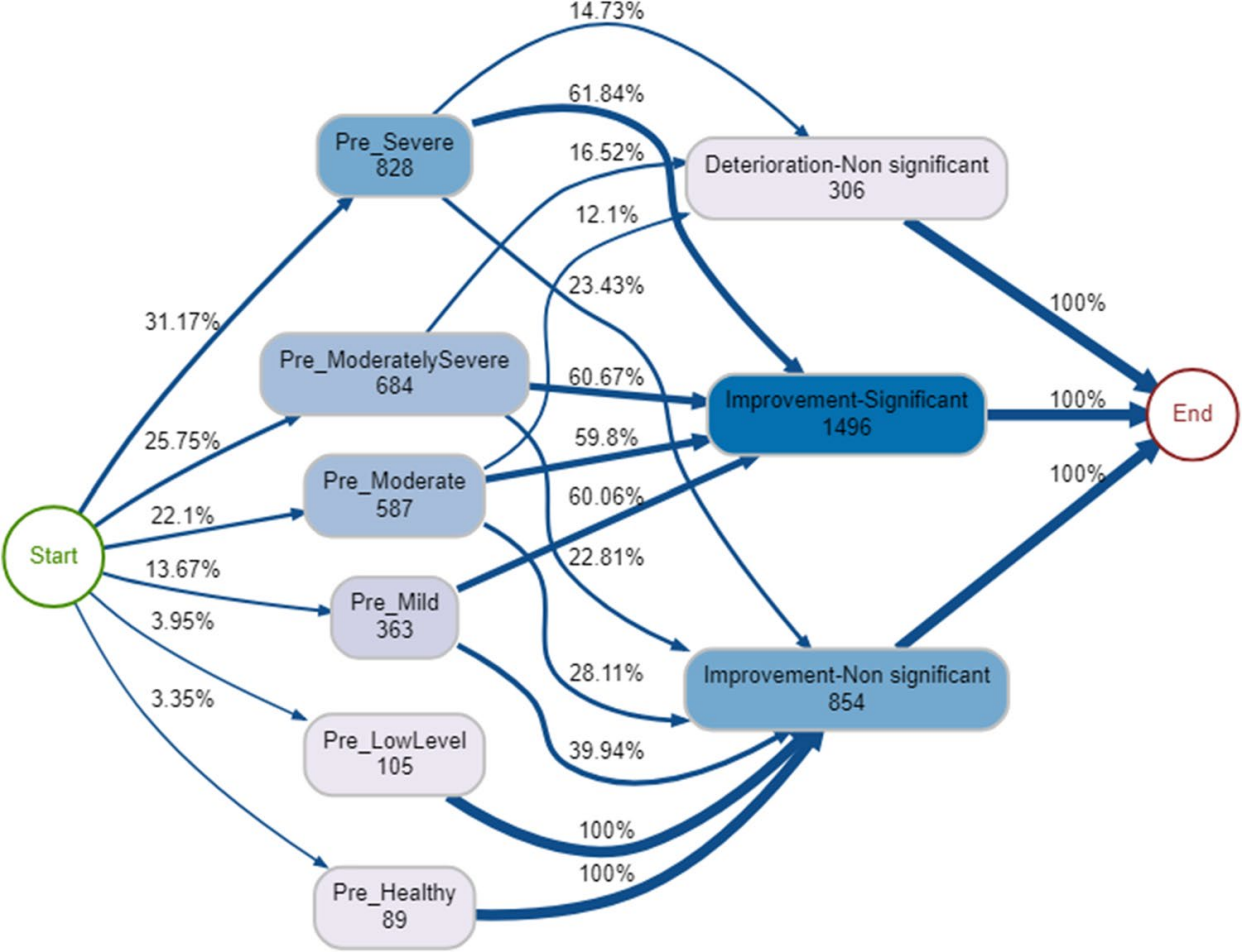
Process map for pre-therapy psychological distress → pre-post-therapy psychological distress change (90% therapy episodes shown)

In a model representing 90% of therapy episodes, the vast majority improve significantly from the beginning to end of therapy (Fig. 3). Of the clients shown in Fig. 3, all of those in the pre-healthy and pre-low bands improved non-significantly. Approximately 15% of those who started off in the pre-severe category shown in Fig. 3 deteriorated non-significantly. The most common single pathway was pre-severe → improvement significant (*n* = 512, 17.5%). Significant deterioration was also a potential outcome, but this was not included in Fig. 3 as it m, represented4.2% of the service(*n* = 123 episodes). The least common pathway through the service included those who started off in a healthy band and deteriorated significantly by the end of therapy (*n* = 8).

**Fig. 4:**
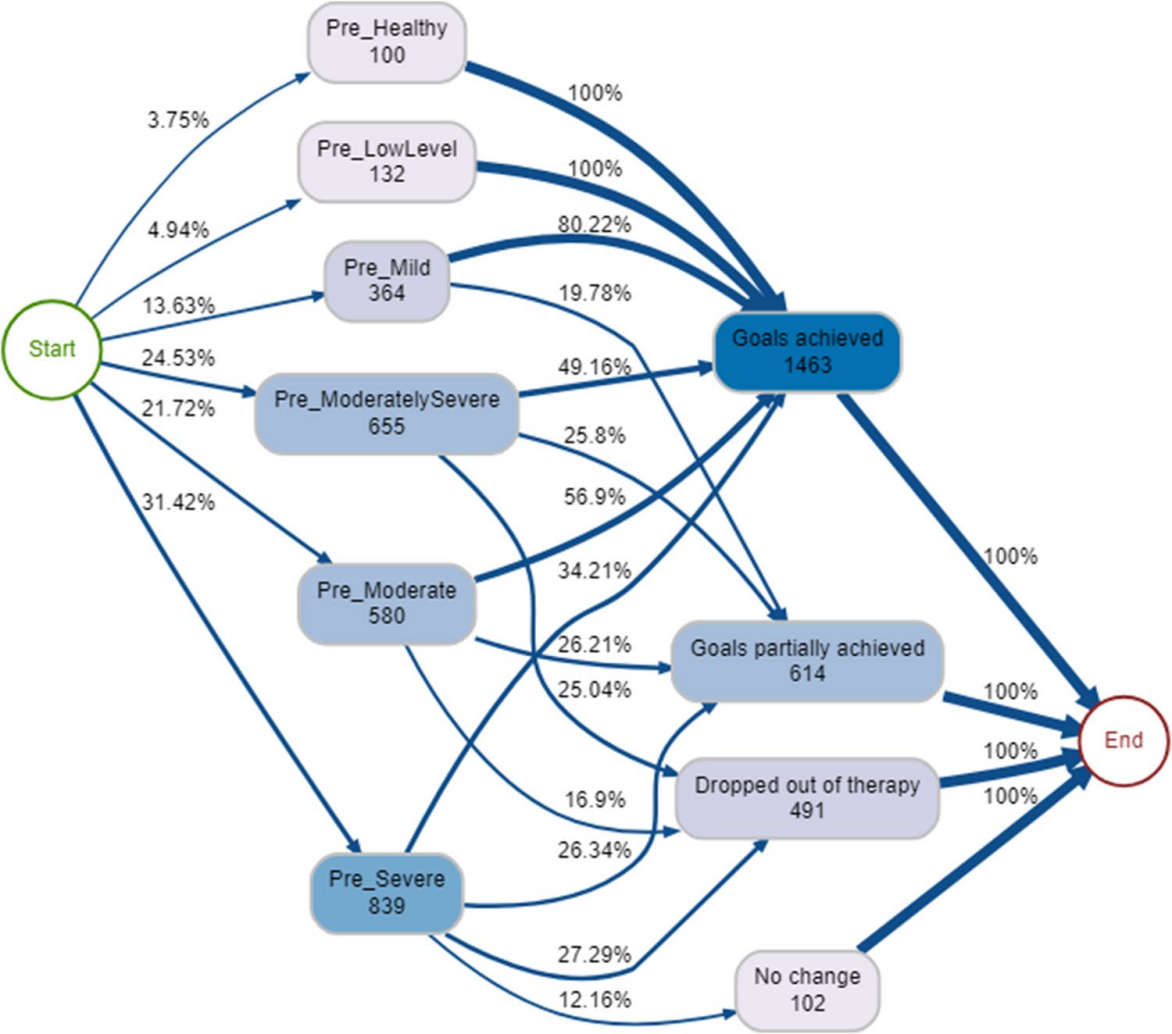
Process map for pre-therapy psychological distress → nature of discharge (90% therapy episodes shown)

Across all pre-therapy psychological distress bands, the majority achieved or partially achieved their goals, especially those who started in the least severe bands (Fig. 4). As psychological distress severity increased, the proportion of clients dropping out also increased (Fig. 4). For example, between 10–13% of those in the lower bands (healthy, low level and mild) dropped out, but this increased to between 15–27% for those in the higher bands (moderate, moderately severe, and severe). The most common single pathway through the service was pre-moderate → goals achieved (*n* = 330, 11.3%). The least common pathways for clients included those who started in the least severe psychological distress bands and did not change or dropped out of therapy.

#### Process mining: number of sessions

**Fig. 5:**
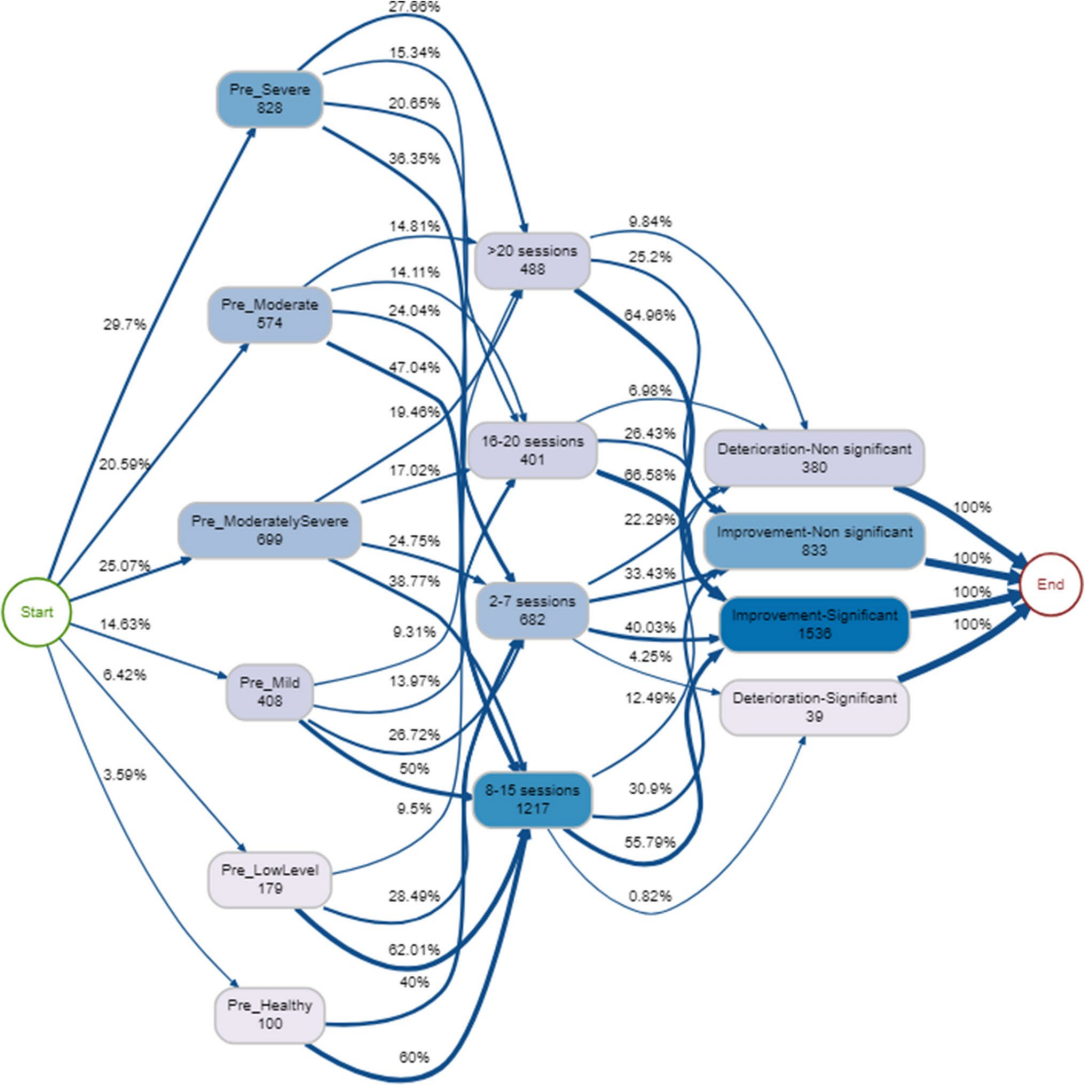
Process map for pre-therapy psychological distress → total number of sessions → pre-post psychological distress change (95% therapy episodes shown)

For the majority of therapy episodes (95%), severity of pre-therapy psychological distress is related to the total number of sessions, with more severe pre-therapy psychological distress bands requiring more sessions (Fig. 5). In addition, there is a weak positive correlation between number of sessions attended and pre-therapy psychological distress score (*r* = 0.2, *p* < 0.001). As a trend, those who have had 8 or more sessions tend to have more positive outcomes, with over half improving significantly (Fig. 5). The most common outcome was significant improvement regardless of pre-therapy psychological distress band or number of sessions (Fig. 5). As session lengths increased from 2–7 to 8–15, and again to 16–20, the relative frequency of significant improvement increased but this was not observed when therapy length went beyond 20 sessions (Fig. 5). Overall, a small proportion of service users attended group sessions only (*n* = 91, 3.1%) and these tended to be clients with a higher degree of psychological distress. Similarly, a small proportion of clients attended a mix of individual and group sessions (*n* = 163, 5.6%), typically those with a moderate, moderately severe and severe psychological distress. Most of the clients that attended a mix of individual and group sessions improved either significantly (48%) or non-significantly (31%). Overall, the most common pathway through the service was pre-severe → 8–15 total sessions → improvement significant (*n* = 197, 6.7%). The least common pathways were those who started with mild, low and moderate psychological distress, attended 16 or more sessions in total, and deteriorated significantly.

#### Process mining: missed sessions

**Fig. 6:**
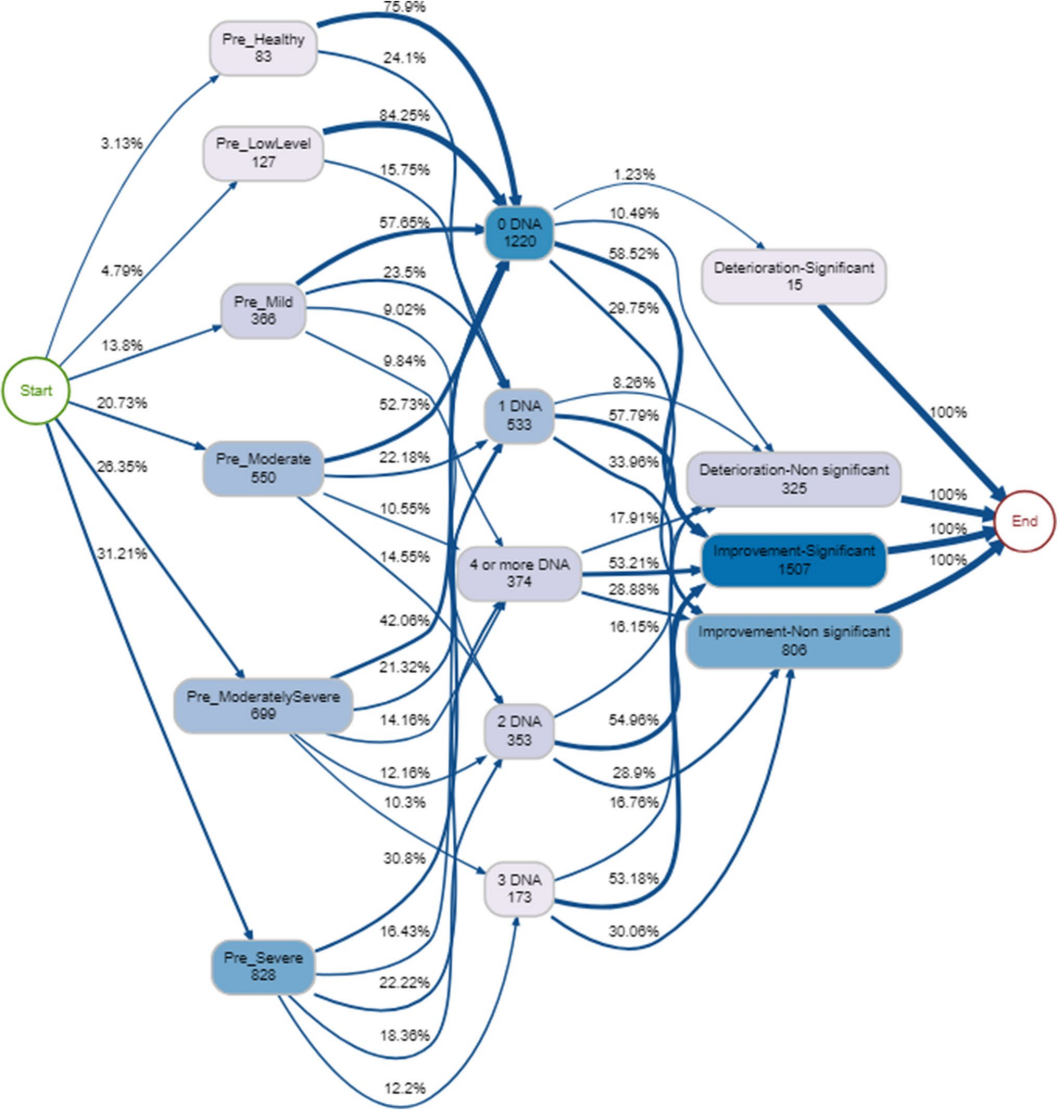
Process map for pre-therapy psychological distress → DNAs → pre-post psychological distress change (90% of therapy episodes shown)

At least three quarters of those with ‘healthy’ or ‘low’ psychological distress when starting therapy had 0 DNAs (Fig. 6). Those who were classed at baseline as having moderate, moderately severe, or severe psychological distress had more DNAs compared to clients with lower pre psychological distress (Fig. 6). In a model accounting for 90% episodes and regardless of DNAs, over half improved significantly post therapy (Fig. 6). The four most common pathways through the service were those that started had 0 DNAs and significantly improved, and started in the moderate (*n* = 192), moderately severe (*n* = 190), severe (*n* = 179) and mild (*n* = 126) bands. One common pathway which does not fit with this trend included those who stared as severe, had 4 or more DNA and improved significantly (*n* = 110, 3.8%). The least common pathways were those who started as pre-healthy/ low, had anywhere from 0 to 4 or more DNAs and deteriorated.

**Fig. 7:**
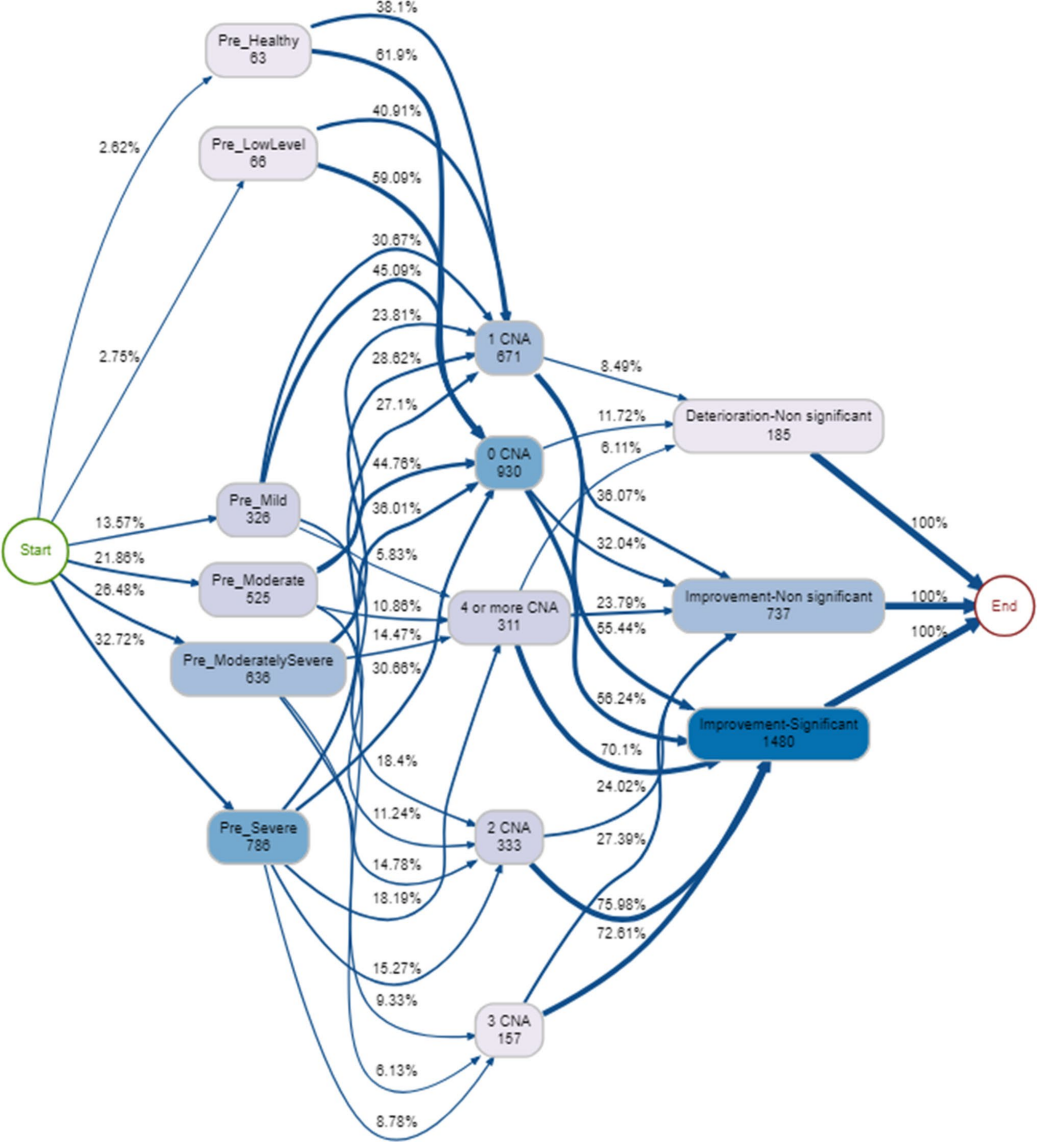
Process map for pre-therapy psychological distress → CNAs → pre-post psychological distress change (80% of therapy episodes shown)

In a model accounting for approximately 80% of therapy episodes (Fig. 7), around 60% of those starting with healthy or low psychological distress had no CNAs, with the remainder having only one CNA. Generally, those with more severe psychological distress had more CNAs (Fig. 7). Looking at 80% of episodes (Fig. 7), at least half improved significantly regardless of the number of CNAs. The most common pathway through the service was pre-moderate → 0 CNA → improvement significant (*n* = 151, 5.2%). Similar to the pathway that included DNAs, another common pathway was pre-severe → 4 or more CNAs → improvement significant (*n* = 108, 3.7%). The least common pathways were those that started as pre-healthy/ mild, cancelled 2 or more appointments and deteriorated.

## Discussion

Process mining provided important insights into the NHSCT PTS functioning by looking at pre-therapy psychological distress severity, missed appointments, and number of sessions attended and how these factors relate to client outcomes. Some clients had very low psychological distress scores at the beginning of therapy and thus it is unlikely these individuals would have significant improvements in outcomes. In general, outcomes were better for clients with fewer cancelled or missed appointments. Pre-therapy psychological distress scores could be a useful factor to consider at assessment for estimating therapy duration, as those with higher scores typically require more sessions. Additionally, the results of this study suggest that RCI appears to be a more valid indicator of therapeutic change in more severe clients, as it detects improvement in symptoms whereas band change does not.

### Process mining approach

Process mining as a whole systems approach is a valuable tool which can be used to understand a healthcare service. It is important that data analysts work closely with healthcare professionals to understand and interpret results. Overall, process mining has some limitations such as the use of taxonomies, events and categorical variables. However, categorical data are useful for clinicians to help decide which individuals to apply policy changes to. Process mining could be a useful precursor to other analytics such as machine learning algorithms which lack transparency, often do not portray system understanding and may be a black box. Process mining visualisations could be help end users understand the relationships and can even be used for feature engineering, providing helpful information for feature selection as opposed to relying solely on a feature importance metric. Linking process mining and machine learning could allow a greater understanding of algorithms where results are more easily interpretable by humans. In mental health, process mining could be useful to explore common routes, whilst ensuring conformance checking by comparing the ideal pathways to real processes. It is also a helpful approach to identify edge cases for those individuals who do not follow a typical pathway through the service.

### Psychological Therapies Service (NHSCT PTS) evaluation

Overall, roughly 1 in 8 clients that presented to the service had a healthy or low psychological distress score at initial assessment, which means they fall below the clinical cut-off point (psychological distress score < 10). Those who begin therapy in the ‘healthy’ band cannot significantly improve and similarly those in the ‘low-level’ band are very unlikely to make significant improvements. It is important for the NHSCT PTS to consider whether to allocate resources to these clients and whether there are other issues that are not measured or disclosed that are relevant. However, it is also possible that for some clients the psychological distress metric (CORE) [43] is not suitable to measure symptom specific problems. Therefore, it may be more useful to look at CORE scores in conjunction with a symptom specific measure (such as Beck's Depression Inventory for those presenting with depression) to assess suitability for therapy. Clinical characteristics such as severity of emotional distress have been found to be more predictive of psychological therapy non-attendance compared to socio-demographic characteristics [17]. Approximately 30% clients (*n* = 839) started therapy with ‘severe’ psychological distress and just over a third of these clients (*n* = 270) remained in ‘severe’ psychological distress category post therapy. While over 60% of those in the pre-severe category improved significantly following therapy, this cohort also had the highest proportion of dropouts in the service (~ 30%), thus it is important to allocate resources to improve the outcomes for these individuals specifically. Many client and therapist reasons for dropping out of therapy have been cited in the literature. A recent review reported that mental health professionals believed the most common reasons for dropout were that clients were not happy with the treatment offered or that they did not see improvements in their mental health [44]. Clients have reported similar reasons, including that in some cases they were dissatisfied with therapy or the professional who they were allocated to and also circumstantial reasons such as financial costs or difficulties with child care [45, 46]. However, it may be that individuals who drop out feel that they have got what they needed from therapy and felt they have achieved their goals [45].

Missed appointments (CNAs and DNAs) were investigated separately to see if there were any differences in outcomes for those who cancelled appointments, versus those who did not show up with no prior notice. Clients with 0 DNA and 0 CNA improved in ~ 80% of cases and over half of those with 1 or more DNA/ CNA significantly improved. Overall, this suggests that low levels of both DNAs and CNAs are associated with better outcomes. This is in line with therapist expectations and supports the assumption that fewer cancelled appointments will result in more significant improvements. It is believed that poor attendance may serve as a proxy for client non-engagement [47, 48]. Hence the findings are in line with previous studies demonstrating the mediating role that engagement plays in therapeutic outcomes [49]. Client engagement in psychotherapy is determined by a number of factors including clients’ motivation, client-therapist relationship, effort put in by the client to attend sessions, and participation in treatment both during and outside therapy sessions [48].

Typically, those with more severe psychological distress at assessment are deemed more likely to require a higher number of therapy sessions compared to those in lower psychological distress bands. The results of the present study support this, and thus pre-therapy psychological distress scores could be useful in making predictions on therapy duration and may help with caseload planning and service management regarding resource allocation. For clients that did present to the service with more severe psychological distress and attended fewer sessions than expected, this was mainly due to clients selecting to drop out of the service. For the small proportion of clients that started therapy with mild, low, moderate psychological distress, attended 16 or more sessions and deteriorated significantly it is possible that the NHSCT PTS was not suitable and alternative treatments may work better for these individuals or that adverse life events co-occurred during the course of treatment.

A recent study looked at treatment outcomes for patients that used IAPT services and found that small changes in clinical practice had positive effects on patient outcomes [15]. Specifically, the authors noted that the delivery of more sessions at more frequent intervals reduced cancellations and were associated with better outcomes in IAPT services [15] which is in keeping with the results of the present study. Going forward the service should reflect on and devise strategies to increase engagement, for example, with the small number (*n* = 123) of clients that deteriorated significantly and attended fifteen or fewer therapy sessions (*n* = 96, 78%). Keeping clients engaged and attending a sufficient number of therapy sessions could be important to improving outcomes. The NHSCT PTS staff had not previously considered pre-therapy psychological distress scores as a predictor of future engagement. However, the present results demonstrate that baseline psychological distress scores could be used to identify ‘at risk clients’ allowing clinicians to undertake early mitigation or even potentially prevent engagement issues from arising in the first place. Addressing DNAs requires meaningful compassionate engagement with the client. A variety of strategies could be used, for example those outlined in the following section.

### Policy and practice recommendations

We propose a series of general policy and practice recommendations, based on the results of this study, given some clients that attended NHSCT PTS had CORE scores below the clinical cut-off and pre-therapy psychological distress scores correlated to the number of sessions.

1. A small percentage of clients entered the service with low pre-therapy psychological distress scores. For these clients, consider administering symptom specific psychometric measures depending on the presenting problem, alongside CORE. Additionally, exercise clinical judgement as to whether psychological intervention is warranted, where alternative treatment options may be more suitable. This is because roughly 1 in 8 clients had a healthy or low psychological distress score at initial assessment, which means they fall below the clinical cut-off point. However, it may be the case that CORE wasn’t a suitable measure for their presenting problem(s).
2. There was a weak positive correlation between the number of sessions attended and pre-therapy psychological distress score (r = 0.2, *p* < 0.001). Thus, pre-therapy psychological distress scores are indicative of therapy duration.This could be useful in terms of the number of therapy sessions which may assist in resource allocation and caseload planning.
3. In the present study, clients with fewer missed and cancelled appointments had better outcomes. Thus, it is important to devise strategies to increase client engagement and attendance in therapy. In the literature, it was reported that delivering sessions at more frequent intervals reduced the number of cancellations. To reduce missed appointments, specific strategies could be used such as trauma-informed support, including peer support, preparation work, reminders, transport and childcare support if necessary, and prioritising access for these clients with flexible appointments, and telephone or text reminders.

Future work should be carried out to further strengthen the results and these recommendations, for example, exploring reasons why people with healthy CORE scores are entering the system. The need to understand factors that influence successful outcomes, and to gather and analyse evidence to best use resources most effectively, is now more pressing than ever. During the pandemic, 95% of sessions at the NHSCT PTS were delivered via telephone or video conferencing software. Anecdotally, clinicians have reported that attendance has improved and missed appointments have nose-dived. Future work can confirm this and analyse the differences in face-to-face versus teletherapy in the NHSCT PTS once sufficient data are available.

### Limitations

The process mining analysis in this study provides a high level overview which doesn’t encompass all potential client pathways, but instead explores the most common pathways through the service. This study is largely exploratory and only looks at a subset of information gathered about clients who attended the NHSCT PTS and a similar methodology should be repeated with data from other services. To allow for process mining to be carried out, numerical variables were transformed to categorical variables which could result in loss of information. In addition, data was collected exclusively in Northern Ireland and therefore may not be broadly applicable to services in other regions. The majority of service users in this study were female (~ 60%) which is typical for clients availing of psychological therapy however, the dataset did not specify those who identify as LGBTQ + , which should be considered in data reporting going foward. The interpretations from process mining should be applied differently at a service level than on a case-by-case basis given the gender imbalance.

## Conclusion

The goals of this study were to explore key insights for practitioners and clinicians gained from process mining of NHSCT PTS data and examine whether process mining could be used to refine and improve mental health services. To the best of our knowledge, this was the first study to apply process mining techniques to data from a mental health service. This work was novel in that it integrated mental health domain knowledge within the process mining pipeline and involved data analysts working closely with mental healthcare providers at different levels (consultants and researchers from NHSCT PTS), not just academics. Process mining could be used for service improvement and a number of policy and practice recommendations have been proposed. In addition, future work will involve exploring least common pathways to identify areas that may be overlooked. This study highlighted a number of key points. Firstly, routinely collecting data is crucial as it can be exploited in novel ways, such as the application of process mining to a new domain. Secondly, it is important to consider evaluation from the outset and collect data accordingly. For example, for process mining from a clinician perspective it would be useful to plan to measure features and key events, start to form links with data analysts and consider methodologies beyond typical research, audit, service evaluation and quality improvement approaches. Finally, adopting an “open” approach to see what emerges from the data as opposed to, for example, testing hypotheses or making assumptions would be useful for generating new insights. This study concludes that process mining is useful in health services such as NHSCT PTS to provide information to inform caseload planning, service management and resource allocation, with the potential to improve clients’ health outcomes.

## Data Availability

The client data analysed during the current study are not publicly available as ethical approval allowed for the data to be shared with the research team only.
